# How coronal alignment affects distal femoral anatomy: an MRI-based comparison of varus and valgus knees

**DOI:** 10.1186/s13018-019-1133-x

**Published:** 2019-04-02

**Authors:** Daniel A Cohen, Ali C Gursel, Adrian K Low

**Affiliations:** Specialty Orthopaedics, Norwest Private Hospital, G18/11 Norbrik Drive Bella Vista, Sydney, NSW 2153 Australia

**Keywords:** Valgus knee, Lateral femoral condyle, Femoral component rotation, Posterior condylar axis, Epicondylar axis

## Abstract

**Purpose:**

In contemporary total knee arthroplasty (TKA), most often, the goal is to align the femoral component to the epicondylar axis (EA). The posterior condylar axis (PCA) is easier to define than the EA, and thus the relationship of PCA to the EA is then used instead to align the femoral component to the EA. However, the relationship of PCA to EA is not constant and has been reported to differ between varus and valgus knees and with increasing deformity. The aim of this large MRI-based study was to evaluate the relationship between PCA and EA with varying coronal deformity especially with increasing valgus deformity.

**Methods:**

EA, PCA, AP (Whiteside’s line) and the mechanical axis were obtained from 474 magnetic resonance imaging (MRI) scans used to create patient-specific instrumentation (PSI) for the Biomet Signature (Warsaw, NJ) system.

**Results:**

The relationship of EA relative to the PCA showed considerable heterogeneity in both varus and valgus groups. In the valgus group, there was statistically greater external rotation (*P* < 0.05) of the EA from the PCA with a mean of 2.52° (range − 1.9° to 6°) compared to the varus group with a mean of 2.03° (range − 3.9° to 6.9°). This relationship did not significantly change with increasing severity of coronal malalignment.

Externally rotating the femoral cutting guide by 3° from the PCA, 11% (42 of 382) of varus knees would lie outside of ± 3° from EA. In valgus knees, externally rotating the femoral cutting block by 3° or 5° from the PCA, 6.5% (6 of 92) and 33.7% (31 of 92) of knees, respectively, would lie outside of ± 3° from EA.

**Conclusion:**

The relationship of PCA to EA is heterogeneous and is not altered significantly with increasing valgus coronal deformity. External rotation beyond 3° from PCA in valgus knees may lead to significant femoral component malrotation in a large proportion cases.

## Introduction

Successful total knee arthroplasty (TKA) relies on accurate placement of femoral and tibial components in the sagittal, coronal and axial plane to restore the mechanical axis of the limb and soft tissue tension within the knee. With respect to the axial rotation of the femoral component, various axes including the epicondylar axis (EA), the anteroposterior axis or Whiteside’s line (AP), and the posterior condylar axis (PCA) assist in correct positioning [1–3].

In contemporary TKA, most often, the femoral component is aligned parallel to the EA. The EA is generally considered the axis about which flexion and extension occurs, and rotation parallel to the EA creates a balanced flexion gap relative to a perpendicular tibial cut. However, this axis is difficult to define accurately intraoperatively [4]. In contrast, the posterior femoral condyles and therefore the PCA are better defined. As such, the relationship of the PCA to the EA is used as a surrogate to determine the axial rotation of the femoral component.

Studies have shown that the PCA is on average 3° internally rotated relative to the EA in normal knees [5]. Therefore, when conventional alignment jigs are used, the femoral cutting block is usually externally rotated from the PCA by 3° to align the femoral component with the EA. In valgus knees, it is common to externally rotate the cutting block to 5° to compensate for lateral condyle hypoplasia. This has been shown to exist in valgus arthritic knees in a cadaver study [6]. However, more recent MRI studies have shown that there is considerable heterogeneity between the relationship of the EA to the PCA and that the PCA vs. EA relationship is not as deranged as previously hypothesised [7, 8].

The aim of the current study was to determine the impact of increasing coronal deformity, specifically valgus deformity, on the relationship between the PCA and EA utilising a large series of MRIs performed on arthritic knees.

## Methods

We analysed 474 consecutive MRIs from 398 patients who had undergone total knee replacement by 2 surgeons. The purpose of the MRIs was to create patient-specific cutting blocks for the Biomet Signature patient-specific system for use during surgery.

Ankle, hip and knee MRI of the affected limb were performed according to the Biomet Signature protocol (Warsaw, NJ). Using Mimics software (Materialise, Leuven, Belgium), the MRI sequences were converted into representative three-dimensional models of the knee. From these images, the Signature software engineers determined overall limb alignment, EA, PCA, Whiteside’s line and the valgus cut angle, defined as the difference between the anatomical and mechanical axis of the femur. This data for each individual patient is available for their surgeon from the Signature Online Management System. No changes in these parameters were made by the primary surgeon.

**Fig. 1:**
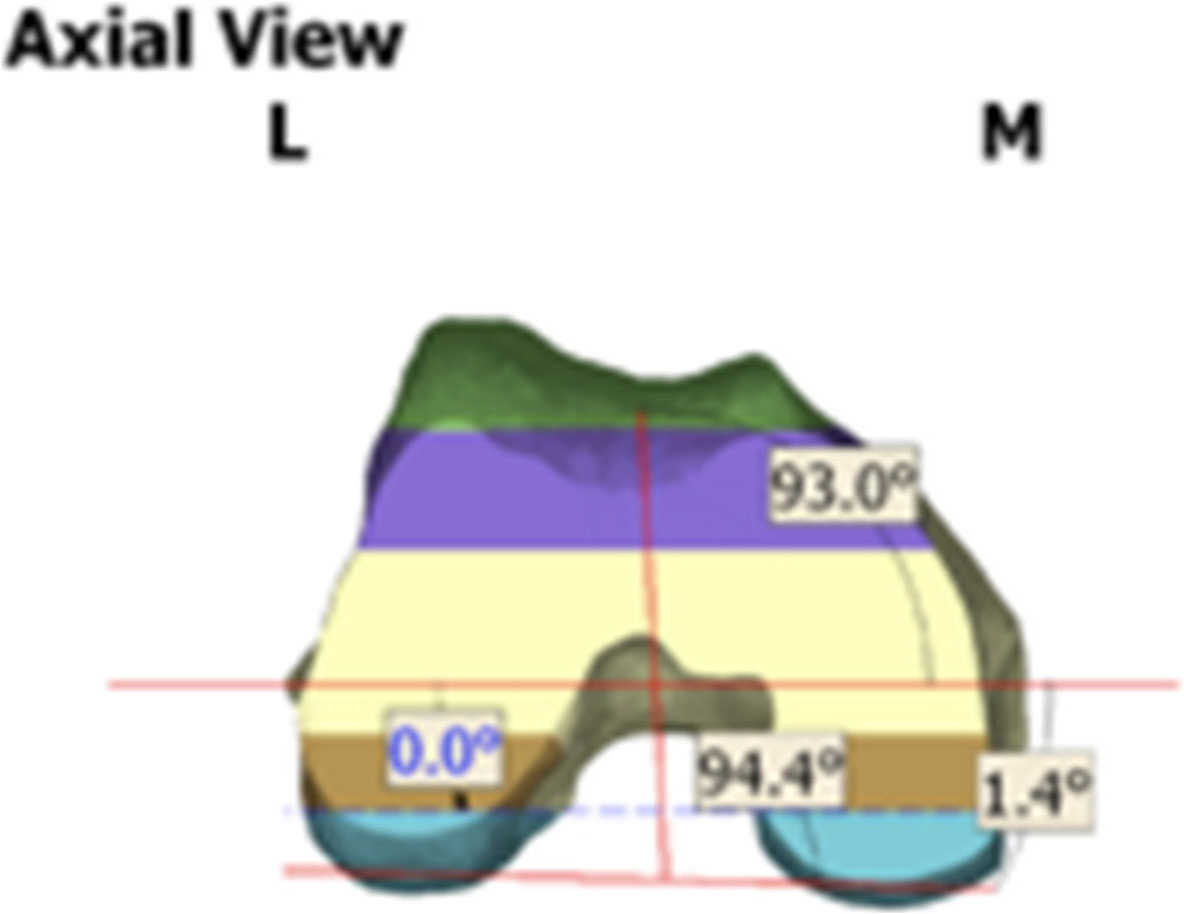
Biomet Signature graphic comparing axes

The mechanical axis of the limb was determined on axial cuts of the hip and ankle (Fig. 1). The femoral head and ipsilateral talus were fit within a circle and a point corresponding to the centre chosen. The line connecting these points was used to determine the mechanical axis of the limb. This is a non-weight-bearing mechanical axis.

The normal mechanical axis of the limb is defined by a line passing from the centre of the femoral head to the centre of the talus. This line should pass through the centre of the knee. Valgus malalignment is defined by a mechanical axis which passes lateral to the centre of the knee while varus malalignment is defined by a mechanical axis which passes medial to the centre of the knee.

The epicondylar axis (EA) was defined by a line between the most prominent point on the lateral femoral epicondyle and the sulcus of the medial femoral epicondyle. These points correspond to the origin of the respective collateral ligaments. Whiteside’s line was defined by a line connecting the deepest point of the trochlea groove and a point at the top of the femoral notch. The PCA was defined by a line tangent to the most prominent point of the posterior femoral condyles.

Age and operative side was recorded for each patient. The direction (varus or valgus) and degree of coronal deformity, the relationship between the PCA vs. EA, AP vs. EA and AP vs. PCA were obtained from the surgical planning template in the Signature Online Management System. To study the effect of increasing coronal deformity on the relationship of PCA vs. EA, we divided the valgus and varus groups into 0–5°, > 5–10° and > 10° of deformity.

Statistical significance, defined as a *P* value < 0.05 was determined using Student’s *t* test, and descriptive measures including mean, median, standard deviation and confidence intervals were ascertained.

## Results

There were 382 knees in 313 patients (mean age 65.7) in the varus group and 92 knees in 85 patients (mean age 67.1) in the valgus group. The mean non-weight-bearing coronal deformity was 6.2° (95% CI 5.92–6.57) in the varus group while the valgus group average deformity was 5.4° (95% CI 4.62–6.18) (Table 1).

**Table 1:** Group characteristics

	Varus	Valgus
*N*	382	92
Avg. age	65.7	67.1
Avg. coronal alignment	6.1	5.4

**Fig. 2:**
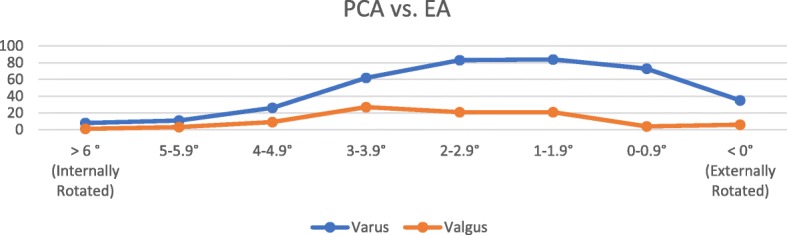
Graphical distribution of PCA vs. EA

In the valgus group there was statistically greater external rotation (P < 0.05) of the EA from the PCA with a mean of 2.52° (median 2.7°, 95% CI 2.21–2.834, range − 1.9° to 6°) compared to the varus group with a mean of 2.03° (median 1.95°, 95% CI 1.86–2.201, range − 3.9° to 6.9°) (Table 2 & Fig. 2).

**Table 2:** Relationship of distal femoral axes

	Varus	Valgus	Significance
Mean PCA vs. EA	2.03 (95% CI 1.86–2.20)	2.52 (95% CI 2.21–2.83)	*P < 0.05*
Mean PCA vs. AP	94.94 (95% CI 94.7–95.2)	95.78 (95% CI 95.3–96.3)	*P < 0.05*
Mean EA vs. AP	92.93 (95% CI 92.7–93.2)	93.27 (95% CI 92.8–93.8)	*P* > 0.05

Subgroup analysis of patients with larger coronal deformity for both varus (0–5°, > 5–10° and > 10°) and valgus (0–5°, > 5–10° and > 10°) showed no statistical difference (*P* > 0.05) in the EA vs. PCA relationship than those with lesser coronal deformity (Tables 3 and 4).

**Table 3:** Varus subgroup analysis

	0–5°	> 5–10°	> 10°
*N*	166	176	40
Mean	2.16	2.02	1.53
CI	1.91–2.41	1.76–2.28	0.98–2.08
SD	1.642	1.735	1.792

**Table 4:** Valgus subgroup analysis

	0–5°	> 5–10°	> 10°
*N*	54	31	7
Mean	2.48	2.42	3.34
CI	2.06–2.90	1.90–2.96	2.88–3.81
SD	1.581	1.494	0.624

With respect to the relationship of the AP vs. PCA axes, in the valgus group, there was statistically greater external rotation of the AP relative to the PCA with a mean 95.78° (95% CI 95.25–96.31, range 87.8° to 100.6°) compared to the varus group with mean 94.94° (95% CI 94.16–94.72, range 85.8° to 104.8°) (Tables 2).

With respect to the AP vs. EA relationship, in the valgus group, the mean external rotation of the AP from the EA was 93.27° (95% CI 92.8–93.8) and this was not statistically significant (*P* > 0.05) from the varus group mean of 92.93° (95% CI 92.7–93.2) (Table 2).

As discussed, in TKA, the aim is to align the femoral component rotation axis to the EA and the acceptable range is within ± 3° of this axis. In varus knees (mean 2.0° ER of EA vs. PCA), if an attempt to align the femoral component parallel to the EA by externally rotating the femoral cutting block 3° is performed, we determined in this series that 11% (42 of 382) of femoral components will lie outside the acceptable range. In valgus knees (mean 2.5° ER of EA vs. PC), if the cutting block is externally rotated by 3° or 5°, then 6.5% (6 of 92) and 33.7% (31 of 92) of femoral components, respectively, will lie outside of the accepted range.

## Discussion

Placement of the femoral component in the arthritic knee requires an understanding of the bony changes that may occur in the distal femur. Cadaver studies have previously shown that changes in the lateral femoral condyle of the valgus knee alter the distal femoral anatomy, specifically leading to derangement of the PCA [6].

In our MRI-based study, we did not find a significant difference in the relationship between PCA vs. EA (2.5° vs. 2.0°) and PCA vs. AP (95.78° vs. 94.94°) in valgus and varus knees respectively. Our results are consistent with other MRI-based studies which have also reported that the bony changes in the valgus knee do not alter the PCA as drastically as once suggested [7, 8]. It is however possible that lateral femoral hypoplasia, that is the posterior as well as the distal surfaces of the lateral condyle are affected, is only seen with larger valgus deformities. In our study, the mean varus deformity was 6.2° and mean valgus deformity was 5.4°. An MRI-based study by Matsuda et al., with an average valgus deformity of 13.3 ± 4.9°, demonstrated a 5° difference in the rotation of the PCA vs. EA compared to varus and normal knees [9]. In our study, we did not find a significant difference in PCA vs. EA between knees with a valgus deformity of < 5°, 5–10° and > 10°. However, we had only 7 knees in the > 10° group.

Alignment of the femoral component in the axial plane parallel to the EA has been recommended as this is considered to be the functional axis about which knee flexion and extension occurs [10–14]. There is considerable variability, even amongst experienced surgeons, in accurately determining this axis intraoperatively [4]. The PCA, on the other hand, is much easier to identify intraoperatively, and as such the relationship of the PCA to the EA can be used to assist in alignment of the femoral component to the EA.

The relationship of PCA to EA, however, is not constant. In our study, there was considerable variation in the relationship between PCA to EA which ranged from external rotation of 2–3° and internal rotation of up to 6° in both varus and valgus knees. Many conventional jig posterior referenced TKA systems recommend external rotation of the femoral cutting jig to 3° in varus knees and 5° in valgus knees relative to the native PCA in order to align the femoral component with the EA. Based on our results, choosing to externally rotate the femoral cutting guide by 3° relative to the native PCA would lead to a rotational malalignment (>3° from EA) in femoral component placement in 11% of varus knees and 6.5% of valgus knees. Externally rotating the femoral cutting guide by 5° from the PCA in valgus knees would lead to a rotational malalignment in 33.7%. A 2010 study using MRI to assess femoral component rotation reported the number of > 3° outliers in conventional jig TKA systems was 22.9% [15].

Malrotation of the femoral component in the axial plane leads to patellofemoral mal-tracking, overtightening of the lateral (excessive ER) or medial (excessive IR) compartments, especially in flexion and thus an overall kinematic mismatch. [4, 9, 16–18] Additionally, femoral component malalignment in the axial plane has been associated with poor patient-reported outcomes, accelerated wear and an increased revision rate [5, 11, 19].

There were limitations to this study. The overall limb alignment was calculated using a non-weight-bearing imaging modality. Use of weight-bearing x-rays would probably accentuate the deformity in many cases. This may affect the validity of our results. Furthermore, it is possible that the posterior aspect of the lateral femoral condyle is only significantly affected with larger valgus deformities. We may not have observed these changes due to the small number of patients in this study with large valgus deformities (> 10°). Finally, determination of the various axes was performed by an unknown number of software engineers based on single points and subject to error. We do not have knowledge of the inter- and intra-observer reliability of these measurements.

## Conclusion

Our study showed that valgus deformity does not significantly alter the relationship of the PCA vs. the EA and AP axis as previous studies suggest. The relationship of the PCA to the EA and AP axes in both varus and valgus deformity is quite heterogeneous and not reliable in isolation for proper femoral component rotational alignment. External rotation beyond 3° from PCA in valgus knees may lead to significant femoral component malrotation in a large proportion cases.

## Abbreviations

AP: Anteroposterior axis
EA: Epicondylar axis
MRI: Magnetic resonance imaging
PCA: Posterior condylar
TKA: Total knee arthroplasty
